# Transcriptome characterization and polymorphism detection between subspecies of big sagebrush (*Artemisia tridentata*)

**DOI:** 10.1186/1471-2164-12-370

**Published:** 2011-07-18

**Authors:** Prabin Bajgain, Bryce A Richardson, Jared C Price, Richard C Cronn, Joshua A Udall

**Affiliations:** 1 Plant and Wildlife Science Department, Brigham Young University, Provo, UT 84602, USA; 2 Rocky Mountain Research Station, USDA Forest Service, Provo, UT 84606, USA; 3 Computer Science Department, Brigham Young University, Provo, UT 84602, USA; 4 Pacific Northwest Research Station, USDA Forest Service, Corvallis, OR 97331, USA

## Abstract

**Background:**

Big sagebrush (*Artemisia tridentata*) is one of the most widely distributed and ecologically important shrub species in western North America. This species serves as a critical habitat and food resource for many animals and invertebrates. Habitat loss due to a combination of disturbances followed by establishment of invasive plant species is a serious threat to big sagebrush ecosystem sustainability. Lack of genomic data has limited our understanding of the evolutionary history and ecological adaptation in this species. Here, we report on the sequencing of expressed sequence tags (ESTs) and detection of single nucleotide polymorphism (SNP) and simple sequence repeat (SSR) markers in subspecies of big sagebrush.

**Results:**

cDNA of *A. tridentata *sspp. *tridentata *and *vaseyana *were normalized and sequenced using the 454 GS FLX Titanium pyrosequencing technology. Assembly of the reads resulted in 20,357 contig consensus sequences in ssp. *tridentata *and 20,250 contigs in ssp. *vaseyana*. A BLASTx search against the non-redundant (NR) protein database using 29,541 consensus sequences obtained from a combined assembly resulted in 21,436 sequences with significant blast alignments (≤ 1e^-15^). A total of 20,952 SNPs and 119 polymorphic SSRs were detected between the two subspecies. SNPs were validated through various methods including sequence capture. Validation of SNPs in different individuals uncovered a high level of nucleotide variation in EST sequences. EST sequences of a third, tetraploid subspecies (ssp. *wyomingensis*) obtained by Illumina sequencing were mapped to the consensus sequences of the combined 454 EST assembly. Approximately one-third of the SNPs between sspp. *tridentata *and *vaseyana *identified in the combined assembly were also polymorphic within the two geographically distant ssp. *wyomingensis *samples.

**Conclusion:**

We have produced a large EST dataset for *Artemisia tridentata*, which contains a large sample of the big sagebrush leaf transcriptome. SNP mapping among the three subspecies suggest the origin of ssp. *wyomingensis *via mixed ancestry. A large number of SNP and SSR markers provide the foundation for future research to address questions in big sagebrush evolution, ecological genetics, and conservation using genomic approaches.

## Background

*Artemisia *(Asteraceae) is a widely distributed plant genus that is predominantly found in temperate regions of the northern hemisphere. Some species within this genus are valued in medicine, cooking, and forage for livestock [1,2]. Big sagebrush (*Artemisia tridentata*) is recognized for its importance to ecosystem function. Big sagebrush is one of the most abundant, widespread, and ecologically important woody shrub species in the intermountain regions of western North America. This species contains three widespread subspecies (*A. t. *ssp. *tridentata, A. t. *ssp. *vaseyana*, and *A. t. *ssp. *wyomingensis*) that occupy distinct habitats and two less common subspecies (*A. t. *ssp. *spiciformis *and *A. t. *ssp. *xericensis*) [3,4]. Prior to the Anglo-American settlement, big sagebrush was estimated to occupy up to 100 million ha of the western United States, while contemporary estimates have shown that the area has been reduced to approximately 43 million ha [5]. Changes in land use and disturbance regimes (*e.g.*, conversion to agriculture, overgrazing and wildfire frequencies) are major factors in the degradation of these ecosystems. Such disturbances can lead to invasions by cheat grass (*Bromus tectorum*) and other weeds that fundamentally change the wildfire frequency and severely reduce the frequency of sagebrush in ecosystems where it historically dominated [6,7]. Restoration of these ecosystems not only requires replanting of big sagebrush, but the replanting should be carried out with a basis of scientific knowledge. Early efforts toward this goal have been made by Mahalovich and McArthur [8], where the authors outline the importance of seed plantation by geographical distribution of the subspecies. Restoration of sustainable populations necessitates understanding of the local and landscape level genetic structure of natural big sagebrush populations.

Polyploidy and intra- and interspecific hybridization are likely the important factors in big sagebrush adaptation and landscape dominance. Big sagebrush subspecies occupy specific ecological niches: ssp. *tridentata *grows in alluvial flats at elevation typically lower than 1800 m, ssp. *vaseyana *is found in higher altitude uplands at elevations above 1660 m up to timberline, and ssp. *wyomingensis *occupies drier sites with shallow soils [9]. Subspecies *wyomingensis *is universally tetraploid, whereas sspp. *tridentata *and *vaseyana *are typically diploid; although both sspp. *tridentata *and *vaseyana *also include tetraploid populations [4]. Hybridization between ssp. *tridentata *and ssp. *vaseyana *is common under the appropriate ecological conditions. Hybridization among big sagebrush subspecies has been studied using reciprocal transplants, showing that natural selection tends to limit the hybrids of sspp. *tridentata *and *vaseyana *to a zone between the parental subspecies habitat [9,10]. McArthur and Sanderson [4] suggest that hybrid zones could be repositories of genetic variation and gene exchange, and can influence the evolution of big sagebrush.

Though widely acknowledged as an important shrub of the intermountain ecosystem in western North America, limited DNA sequence data has been collected on big sagebrush. A search for *A. tridentata *nucleotide sequences in the NCBI database yielded less than 50 nucleotide sequences. As a genus, *Artemisia *has approximately 3.8 million sequences (~823 Mbp) of which 3.7 million reads are archived in the Sequence Read Archive (SRA), from *A. annua *EST projects [2,11], and an ongoing *A. annua *genome project [11]. *A. annua *is a medicinal herb native to temperate Asia and is not found in the western hemisphere. Sequences of *A. tridentata *are needed to conduct research studies involving phylogenetics, population genetics, and ecological genetics in North American big sagebrush populations. Transcriptome sequencing and annotation, and marker detection within big sagebrush EST sequences will provide a rapid means to satisfy these information needs and set the stage for future studies.

In this study, we characterized the leaf transcriptome of two big sagebrush subspecies, *A.t. *ssp. *tridentata *and *A.t. *ssp. *vaseyana *and compared the resulting ESTs. We also sequenced *A. t. *ssp. *wyomingensis *ESTs to assess SNP distribution in this subspecies compared to *sspp. tridentata *and *vaseyana*. Our objectives were to 1) identify and characterize a large number of expressed genes in *A. tridentata*, 2) detect sequence differences within and between sspp. *tridentata *and *vaseyana *that could be used as markers to further our understanding of adaptive, phenotypic variation within the species, and 3) gain inference into the origins of the tetraploid *A. t. *ssp. *wyomingensis*.

## Results

### EST sequencing, assembly, and characterization

**Figure 1:**
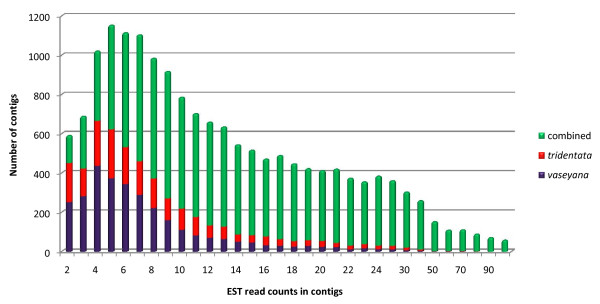
**Histogram of number of EST members in a contig**. Different colors in the bars indicate contigs from the combined assembly composed of ESTs from each subspecies and those derived from ESTs from both subspecies. Contigs with more than 100 EST members are not illustrated.

We created two normalized cDNA libraries from leaf tissues of two subspecies of *A. tridentata*: sspp. *tridentata *and *vaseyana*. Independent sequencing of these cDNA libraries generated 823,392 sequence reads containing 332,578,737 bases of sequence from ssp. *tridentata *and 702,001 sequence reads containing 233,854,535 bases of sequence from ssp. *vaseyana *(Table 1). Assuming a limited amount of sequence divergence between the two subspecies' coding sequence, both sets of ESTs were combined into a single, *de novo *assembly (Table 1). This assembly contained in 29,541 contigs and 275,866 singleton sequences. From the assembled contigs, ESTScan [12] predicted 25,998 (88%) protein coding open reading frames, of which 25,089 (96%) were greater than 200 bp. Some contigs were assembled from reads of a single subspecies. 2,381 contigs were exclusively composed of ssp. *tridentata *reads and 3,137 contigs were exclusively composed of only ssp. *vaseyana *reads (Figure 1). EST read number ranged from 2 reads to 3,161 reads in a contig, with a median of 23 EST reads per contigs. Unless stated otherwise, the combined assembly sequences were used for subsequent bioinformatic analyses. From the remaining, unassembled reads (singletons), ESTScan predicted 136,305 (49.4%) protein coding open reading frames, of which 112,028 (82.2%) were greater than 200 bp.

**Table 1:** Summary of individual and combined *de novo *assembly of the subspecies sequences generated from 454-pyrosequencing

Assembly		Count	Average length	N50 (bp)	Total bases
ssp.	Reads	823,392	403.9		332,578,737
*tridentata*	Singletons	191,745	403.6		77,391,754
	Contigs	20,357	716.0	869	14,587,705

ssp.	Reads	702,001	333.1		233,854,535
*vaseyana*	Singletons	179,189	331.5		59,402,844
	Contigs	20,250	624.0	797	12,641,189

ssp.	Reads	1,525,393	371.3		566,433,272
combined	Singletons	275,866	370.2		102,121,262
	Contigs	29,541	796.0	1,003	23,521,465

**Figure 2:**
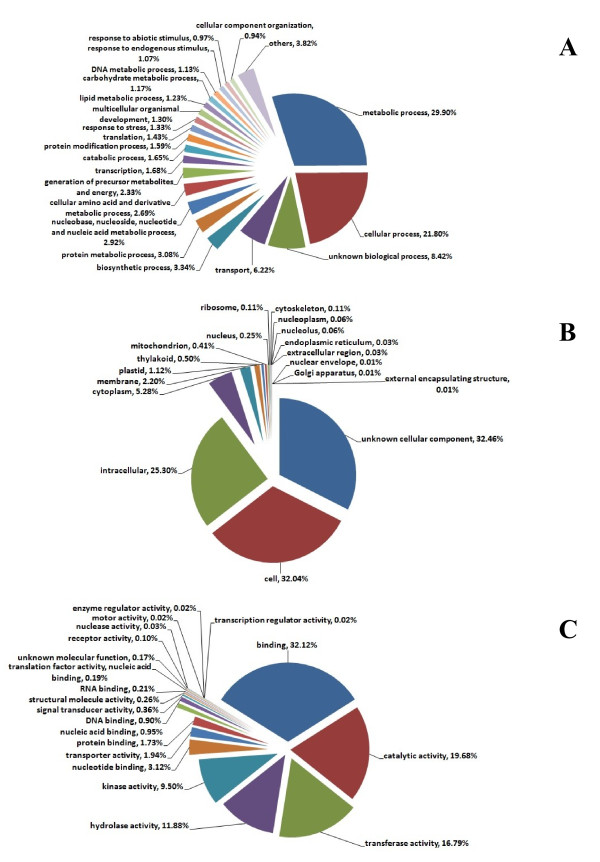
**Distribution of consensus sequences from combined assembly of *Artemisia tridentata *ssp. *tridentata *and ssp. *vaseyana *in three main GO categories**. A: Distribution of GO terms in biological process (BP) category; B: Distribution of GO terms in cellular component (CC) category; C: Distribution of GO terms in molecular function (MF) category. The annotated contigs were passed through GOSlimViewer to obtain a high level summary of functions. The number of contigs annotated to BP, CC and MF categories were 8,144, 10,488, and 14,000, respectively.

Protein homologies between big sagebrush ESTs and the NR protein database were identified using BLASTx. Out of 29,541 assembled contig sequences, 21,436 sequences (72.6%) had significant alignments (≤1e^-15^) and out of 275,866 singleton sequences, 70,836 sequences (25.7%) had significant alignments (≤1e^-15^). Of the contigs with BLASTx hits, 9,696 (45.2%) sequences had their best blast alignment to *Vitis vinifera *and 215 (7.3%) and 80 (0.27%) to *Helianthus annus *and *Lactuca sativa *respectively, both of which are in the Asteraceae. Only fifty-four (0.2%) contigs had their best matches to *Artemisia annua*, owing to the limited number of Artemisia proteins (167) in the NR protein database. The NR BLAST results were utilized by Blast2GO [13] to annotate the EST sequences with GO terms. One or more GO IDs were assigned to 18,397 (62.3%) contigs with a maximum of 21 GO IDs assigned to a single sequence. The distributions of contigs in three, non-mutually exclusive GO categories: biological process (BP), cellular component (CC), and molecular function (MF) were well represented by a diverse set of putative biological functions (Figure 2). In BP category, the most abundant GO term was metabolic process (29.9%), followed by cellular process (21.8%), and unknown biological process (8.4%). In CC category, unknown cellular component was the most abundant (32.5%), followed by cell component (32.0%) and intracellular component (25.3%). Similarly in the MF category, binding was the most abundant category (32.1%), followed by catalytic activity (19.7%), and transferase activity (16.8%). The three groups (BP, CC and MF) are not mutually exclusive; therefore, some contigs were assigned gene ontologies in more than one type of category.

Comparison of the 29,541 contig sequences against the Pfam-A domain database with an e-value cutoff at 1e^-5 ^resulted in 15,812 contigs (53.5%) matching at least one protein domain model. The distribution of the domains ranged from a maximum of 13 domains assigned to the same contig to a minimum of one domain per contig (Additional File 1). The three most common domains were the Protein kinase domain (Pkinase, Pfam ID: PF00069.18), followed by the Protein tyrosine kinase domain (Pkinase_Tyr, Pfam ID: PF07714.10), and the RNA recognition motif domain (RRF_1, Pfam ID: PF00076.15).

### Genes associated with secondary metabolites synthesis in A. tridentata

Big sagebrush leaves are known to synthesize and store large quantities of terpenoids on the epidermal surfaces of glandular leaf trichomes [14]. Therefore, a search was conducted among the annotated contigs to identify putative genes that code for enzymes involved in terpenoid synthesis via the Mevalonic acid (MVA) and 2-C-Methyl-D-Erythritol-4-Phosphate (MEP) biosynthetic pathways [2]. Most of the enzymes involved in these pathways were detected in our annotated contig sequences, and are presented in the additional materials (Additional File 2). Coumarin derivatives are considered as a tool for subspecies identification because big sagebrush subspecies differ in their amount of fluorescence [15,16]. We also searched the annotated contigs for enzymes involved in coumarin biosynthesis. Coumarins in plants are derived via the phenylpropanoid pathway from *p*-coumaroyl CoA [17,18]. At the top of the phenylpropanoid pathway, phenylalanine lyase acts on the substrate L-phenylalanine, and converts it to cinnamate (often, *trans*-cinnamate) which is then oxidized to *p*-coumarate by the enzyme cinnamate 4-hydroxylase. The enzyme 4-hydroxycinnamoyl CoA ligase then ligates an S-CoA group to *p*-coumarate, forming *p*-coumaroyl CoA. We were able to detect putative gene sequences for these three main enzymes involved in coumarin synthesis in our annotated contig dataset. Individual tests of enzymatic substrates and products are needed to confirm their roles in coumarin synthesis within big sagebrush.

### Discovery and frequency of SNPs in ESTs

A large number of resident SNPs were discovered within the accessions examined in this study. A search for SNPs yielded 59,093 ssp. *tridentata *(1 SNP/246 bp) SNPs with an average base coverage of 18 × and 61,028 ssp. *vaseyana *SNPs (1 SNP/207 bp) with an average base coverage of 15×. These putative SNPs may represent a high level of heterozygosity that exists in big sagebrush through its large effective population size and its wind-pollinated reproductive strategy. Future segregation analysis would be needed to ascertain if these putative SNPs are truly two alleles at a single locus or an artifact of the sequencing and/or assembly process. The individual assemblies contained many fewer SNPs than detected in the combined assembly (135,310) prior to filtering those SNPs that were not discriminatory between sspp. *tridentata *and *vaseyana *(*i.e. *nearly homogeneous within a single DNA source, but different between the two DNA sources within a contig).

**Figure 3:**
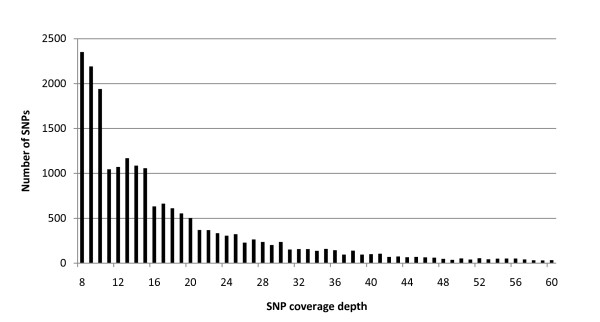
**Distribution of the number of SNPs by read coverage depth**. The average depth of coverage for an SNP was 20×. The numbers of SNPs with read coverage depth of 61 × or higher are not shown.

**Figure 4:**
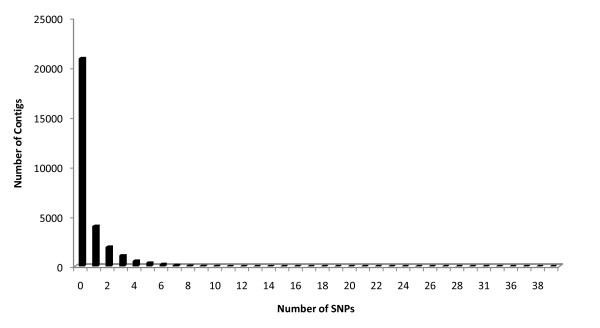
**Distribution of contigs by number of SNPs in a combined assembly of *A. t. *ssp. *tridentata *and ssp. *Vaseyana***. The numbers of contigs with 25 or more SNPs have been grouped together.

When only SNPs that were near homogenous (>90%) within the two accessions were considered, a total of 20,952 SNPs were detected between the sspp. *tridentata *and *vaseyana *when a threshold of 8 × coverage was applied (average of 20 × coverage, Figure 3). The SNP density in the EST sequences between subspecies was 1 SNP/1123 bp. The analysis showed that 8,598 (29%) of the total consensus sequences contained at least one SNP and SNPs per contig ranged from 4,037 contigs with a single SNP to a single contig with 39 SNPs (Figure 4). We chose to exclude SNPs that were less that 90% homogeneous within either accession 1) to provide a reduced set SNPs that could discriminate between the two subspecies and 2) to allow for some degree of sequencing error at the SNP position when there was low sequence coverage. To estimate the effect of SNP heterogeneity within a DNA source, SNPs were also identified with a threshold of homogeneity for SNPs of 99%. This resulted in the detection of 18,173 SNPs indicating that 13% of the SNPs originally detected either ssp. *tridentata *or ssp. *vaseyana *had more than one base at that position albeit at a low frequency. These multiple bases could be the result of heterozygosity, differential expression of alleles, assembly error, or sequencing error. Some of the contigs with high number of SNPs (>13 SNPs) are likely artifacts of applying a single set of assembly parameters to a set of heterogeneous genes and splice forms - each with their own evolutionary history. Collectively, contigs with an abundance of SNPs (>13) are best avoided in future population genetic studies, though individually each contig with a multitude of SNPs might provide an interesting case study of gene or gene family evolution. Contigs with a high number of SNPs have been highlighted in Additional File 3 along with metadata for each contig. Of 20,952 SNPs, 16,317 SNPs were distributed in the putative coding sequence and 4,365 SNPs were in the 5'or 3' untranslated regions. Forty-two percent of the identified SNPs fit within the 20 to 30% range for minor allele frequency, 30% within the 30 to 40% range and the remaining 28% within the 40 to 50% range. As expected, the transition mutations (A/G or C/T) were the most abundant, outnumbering the transversion mutations (A/T, C/A, G/C, G/T) by 3.4 × margin (Table 2). All SNP information of the combined assembly and the sequences with SNPs have been deposited in dbSNP in Genbank. The SNPs are submitted under the handle UDALL_LAB (Genbank: ss252842630 to ss252863866; build B133). Full contig sequences are available upon request.

**Table 2:** Frequency of SNPs between ssp. *tridentata *and ssp. *vaseyana *by mutation types

	SNP type	Count	% Count	% Total
Transitions	C/T	6456	30.81	62.24
	A/G	6586	31.43	
Transversions	A/T	2352	11.23	37.7
	C/G	1533	7.32	
	A/C	2043	9.75	
	G/T	1970	9.40	
	Total	20940	99.94	99.94

### Discovery and frequency of SSRs in ESTs

**Figure 5:**
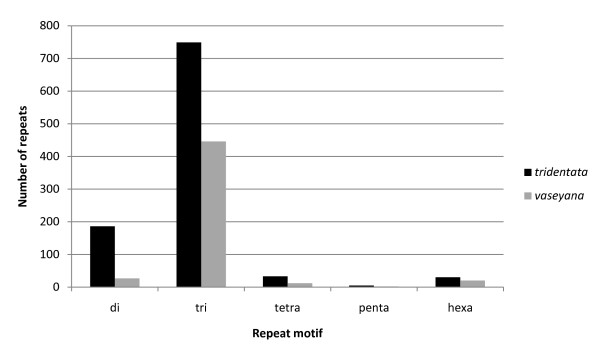
**Frequency and distribution of SSRs in *A. t. *ssp. *tridentata *and *A. t. *ssp. *Vaseyana***.

The individual and combined assemblies of ESTs were used for the SSR analysis. The individual assembly analysis resulted in a total of 908 contigs containing 1,003 SSRs and 466 contigs containing 507 SSRs in ssp. *tridentata *and ssp. *vaseyana*, respectively. Homopolymer SSRs that are reported by default in MISA were not reported because of known limitations of 454 sequencing chemistry. The occurrence and frequency of different SSR motif repeats in EST sequences of the two subspecies were explored (Table 3). Although both subspecies have a similar number of reads, the frequency of each type of SSR motif was almost doubled in ssp. *tridentata *compared to ssp. *vaseyana *(Figure 5). As might be expected from data containing open reading frames, the most common type of repeat was a trinucleotide motif (74.7% in ssp. *tridentata*, 88% in ssp. *vaseyana*), followed by a dinucleotide motif (18.5% in ssp. *tridentata*, 5.3% in ssp. *vaseyana*) and a hexanucleotide motif (3% in ssp. *tridentata*, 4% in ssp. *vaseyana*; Table 3). Repeat motifs unique to each subspecies were also detected. Excluding the counts of SSRs in compound formation, subspecies *tridentata *had 143 unique SSRs and ssp. *vaseyana *had 51 unique SSRs, relative to each other. The most dominant repeat motif overall is AC/GT with a frequency of 15.15% (152 of 1,003) in ssp. *tridentata*, whereas the most dominant repeat motif in both subspecies is ACC/GGT with a frequency of 13.4% (134 of 1003 in ssp. *tridentata*) and 20.7% (105 of 507 in ssp. *vaseyana*). We were unable to detect any CG/GC motif in either subspecies' EST sequences. This could be due to limitations of emPCR used by the 454 sequencing protocol. Additional details about di- and trinucleotide repeat motifs in both subspecies are listed in Additional File 4.

**Table 3:** SSR frequencies of repeat type with repeat numbers in *A. tridentata *ssp. *tridentata *(*A.t.t*.) and ssp. *vaseyana *(*A.t.v.*).

Motif	Repeat number												Total	
	***A.t.t.***	***A.t.v.***	***A.t.t.***	***A.t.v.***	***A.t.t.***	***A.t.v.***	***A.t.t.***	***A.t.v.***	***A.t.t.***	***A.t.v.***	***A.t.t.***	***A.t.v.***	***A.t.t.***	***A.t.v.***
	**5**		**6**		**7**		**8**		**9**		≥**10**			

**Di**	-	-	-	-	104	15	34	2	17	1	31	9	186	27
**Tri**	431	250	186	127	75	35	30	16	10	10	17	8	749	446
**Tetra**	23	10	5	2	3	0	1	0	1	0	0	0	33	12
**Penta**	2	0	2	1	0	1	0	0	1	0	0	0	5	2
**Hexa**	22	16	3	0	2	3	1	1	1	0	1	0	30	20
≥**Hepta**	0	0	0	0	0	0	0	0	0	0	0	0	0	0

**Total**	478	276	196	130	184	51	66	19	30	11	49	17	1003	507
**%**	47.6	54.5	19.5	25.6	18.3	10.1	6.6	3.7	3.0	2.2	4.9	3.8		

In addition to MISA-detected SSRs, a custom Perl script was used to identify putative polymorphic SSRs between ssp. *tridentata *and ssp. *vaseyana *in the combined assembly. Within an assembled contig, the polymorphic SSRs were identified by counting differences in the numbers of repeat motifs during informatic comparison of ssp. *tridentata *sequences to ssp. *vaseyana *sequences. This resulted in detection of 119 polymorphic SSRs in 117 contigs between the two subspecies. Comparing these 119 SSR motif structures to the SSR motif structures identified in individual assemblies, we observed that 111 (93%) SSRs in the combined assembly were found to be identical to those in the individual assemblies and 8 (7%) had a different number of repeats than detected in the individual assemblies.

### SNP and SSR validation

SNPs and SSRs found in the EST assembly were independently validated. SNPs between *A. tridentata *subspecies were directly validated using two different experimental approaches: subsequent Sanger re-sequencing of cDNA amplicons (primers listed in Additional File 5) and by re-sequencing targeted loci by sequence capture. SNPs were not considered validated unless both expected bases were identified in subsequent sequencing efforts and a distinction was made between two different types of validation. Validation Type 1 was where the two different bases identified in the EST assembly were detected at the SNP position (within individual, subspecies, or species). Validation Type 2 was where the two different bases identified in the EST assembly were detected at the SNP position *and *they were consistently different between the two subspecies of *A. tridentata*, as originally detected.

Subsequent Sanger re-sequencing of cDNA amplicons was performed on the same individuals as used for EST sequencing. Analysis of fragment sizes on agarose gel confirmed amplification of all (15 loci) targeted with primers in both subspecies cDNA. Of these loci, 6 were chosen for Sanger re-sequencing. Three SNPs were transitions (1 C↔T; 1 G↔A; 1 T↔C) and three were transversions (2 T↔G; 1 C↔G). The SNP base were validated in cDNA from both subspecies for six of six SNPs (Type 1) and three of six (Type 2), confirming their respective identification within the combined assembly. In the EST assembly, coverage of the chosen SNPs ranged from 9 to 27X and from 20% to 46% in their minor allele frequency. There was no obvious relationship between the amount of EST coverage and SNP validation in this small subset.

Re-sequencing targeted loci by sequence capture was also used to validate SNPs in two distinct individuals of ssp. *tridentata *and two distinct individuals of ssp. *vaseyana *(results for each SNP are listed Additional File 6). 369 ESTs containing 572 discriminating SNPs between the two subspecies were targeted for re-sequencing by sequence capture. These targets were selected based on putative EST function (terpenoid and coumarin biosynthetic pathways) rather than SNP density, thus some contigs had several more SNPs than others. Reads obtained from sequence capture were mapped to their respective reference targets (consensus sequences of contigs from the combined assembly) resulting and 403 SNPs in 134 contigs had capture reads overlapping their respective positions. Both SNP bases were detected at 270 (67%) of the SNP positions (Type 1) and 218 (54%) of the SNP bases were polymorphic (Type 2) between the two independent individuals of ssp. *tridentata *and two independent individuals of ssp. *vaseyana *used for sequence capture. Of the 46% of the SNP positions that did not validate (Type 2), only a single type of base was detected in these four individuals (monomorphic) and the base call always matched one of the expected bases at the SNP. For these monomorphic SNPs, additional sequence coverage of SNP likely would not identify the 'other' base and change our interpretation because these SNPs had an average of 12.6 sequence coverage. 8% of the SNP positions had an additional or third base at the SNP position of a single read in these four individuals.

The low validation rates of SNPs (Type 1: 67% and Type 2: 54%) derived from ESTs could be due to several factors including different genotypes of individual plants, allelic expression biases of sagebrush genes combined with a moderate amount 454 EST sequencing, and errors due to mapping reads to a non-sequenced genome. Different genotypes of individual plants could explain the low SNP validation rate between subspecies. For example, 38% and 10% of SNPs initially detected in our EST assembly were polymorphic between the two individuals of ssp. *tridentata *and polymorphic between the two individuals of ssp. *vaseyana*, respectively. Individual genotypic differences could also explain the 67% level of two-base detection at SNP positions (intra- or inter-subspecies). Of the 403 SNP loci, 16-36% had both bases in individual plants (*i.e. *putative heterozygotes). Thus, it is not too surprising that the four individuals sampled for validation were also coincidently homozygous for many SNPs found in ESTs of a ssp. *tridentata *and in ESTs of a ssp. *vaseyana *individual, particularly if either of the two originally EST-sampled individuals contained a low frequency allele.

SSRs were validated by re-sequencing of Sanger amplicons, 15 loci were selected from the combined EST assembly. Ten of the 15 primer pairs amplified loci in leaf cDNA from both subspecies. Of these 10 loci, 5 loci were selected for Sanger re-sequencing. Re-sequencing of the selected PCR-amplified cDNA sequences confirmed the MISA-detected SSRs (Additional File 5). Variation in repeat length of the re-sequenced loci was verified between subspecies in 3 of the 5 loci. Of these three SSRs, (CTT)_6 _and (GCA)_7 _were bioinformatically detected *a priori *as polymorphic, based on the Perl script parameters, whereas (ATA)_5 _was not, suggesting that the number of bioinformatically identified polymorphic SSRs was an underestimate of the number of truly polymorphic SSRs. We expect that more SSR loci likely exist but they were under the conservative thresholds used in our bioinformatic analysis. The sequence capture experiment also validated a number of SSRs in contig consensus sequences of the combined assembly. Capture targets included 17 putative SSRs, of which 14 had overlapping reads from sequence capture. In every instance, the presence of an SSR was confirmed. Of these 17 SSRs, five SSRs were polymorphic in repeat number, four SSRs contained SNP polymorphisms in one or more repeats, and five SSRs did not have any polymorphisms detected in the sequence capture reads.

### Marker evaluation in genomic DNA

Because of our interest in marker utilization for population genetic studies in genomic DNA (as opposed to ESTs), 15 SSR and 15 SNP primer pairs were evaluated in big sagebrush genomic DNA. Genomic SSR loci were also amplified from the same individuals using the same primers used for SSR validation in cDNA. Fourteen (93%) SSR loci out of 15 SSR loci amplified in both sspp. *tridentata *and *vaseyana *and 11 (73%) SSR loci out of 15 SSR loci amplified in ssp. *wyomingensis*. These 11 primers pairs produced fragments of expected sizes in all three subspecies. Re-sequencing of genomic DNA amplicons for SSR validation was not performed, but we expect that the amplified genomic DNA fragments also contain the targeted SSRs.

Of the 15 SNP primer pairs, 11 (73%) amplified targeted loci in all three subspecies including the five loci used for cDNA SNP validation. The genomic fragments of these five loci were sequenced in two ssp. *tridentata *individuals, three ssp. *vaseyana *individuals and two ssp. *wyomingensis *individuals. For two loci, we observed that both sspp. *tridentata *and *vaseyana *were homozygous at each SNP allele (as expected from the combined assembly) while ssp. *wyomingensis *was dimorphic (i.e. contained both bases). In two different loci, ssp. *wyomingensis *sequences contained a single variant matching either ssp. *tridentata *or ssp. *vaseyana *variant. The remaining SNP remained unconfirmed due to poor Sanger sequencing results. Additional Sanger validation of individual SNP loci would have been an overly laborious process since other sequencing methods exist for validating larger numbers of SNPs (sequence capture and Illumina re-sequencing). Instead of individually genotyping SNP additional loci, genotypic assessment of ssp. *wyomingensis *at putative SNPs loci was determined *en masse *using Illumina sequencing (see below).

### Detection of allelic SNP variants in ssp. wyomingensis

Approximately 2.5 million and 10.5 million Illumina reads were obtained from the Montana and Utah ssp. *wyomingensis *samples, respectively. After trimming the 5' ends of the sequences to remove barcodes, the sequences were aligned to the combined EST assembly (obtained from 454 sequencing of normalized cDNA libraries) as a sequence reference. In the Montana sample, the Illumina reads overlapped 695 SNP positions at a depth of ≥ 20 × with 10% of the reads containing at least one variant. At these SNP positions, both allelic variants (matching the two diploid subspecies) were verified at 251 SNPs. The ssp. *tridentata *base matched at 138 additional SNP positions and the ssp. *vaseyana *base matched at 306 other SNP positions. In the Utah sample, Illumina reads overlapped 1,039 SNP positions at a depth of ≥ 20 × with 10% of the reads containing at least one variant. At these SNP positions, both allelic variants (matching the two diploid subspecies) were verified at 458 SNPs. The ssp. *tridentata *base matched 157 additional SNP positions and the ssp. *vaseyana *based matched at 424 other SNPs positions. Verified SNPs from the Montana sample were distributed among 484 contigs of the combined assembly, and verified SNPs from the Utah sample were distributed among 767 contigs. This variability of SNP detection is not surprising as the leaf tissue samples for Illumina cDNA sequencing were collected in different environments under different conditions and the cDNA fragments were not normalized (like the diploid cDNAs) or modified in any way to alter the association between gene expression levels and number of reads per gene.

### Directional selection in subspecies genes

**Figure 6:**
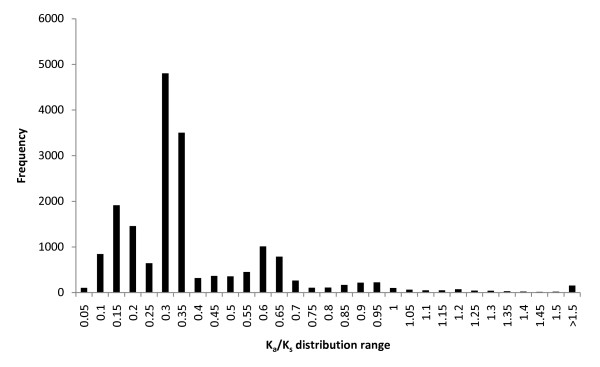
**A histogram illustrating the distribution of the ratio of non-synonymous mutations to non-synonymous sites compared to synonymous mutations per synonymous sites (K**_**a**_**/K**_**s**_**)**.

To detect functional sequence diversity and diversifying selection, putative exon sequences were compared between sspp. *tridentata *and *vaseyana*. Comparison of synonymous base substitutions (K_s_) and non-synonymous base substitutions (K_a_) between the two subspecies would suggest whether these sequences were influenced by stabilizing or diversifying selection. The distribution of the calculated K_a_/K_s _ratios for the contigs was found to be ranging from 0.0132 to 6.4000; however any K_a_/K_s _value greater than 2.5000 was discarded during the analysis assuming that such high values likely resulted from alignment errors. The resultant distribution had a median of 0.2959 (standard deviation = 0.2627). A bootstrap analysis with 146,744 data points at 95% confidence level (α = 0.025) was also performed on the K_a_/K_s _distribution, which resulted in 0.0791 as the lower limit and 1.0880 as the upper limit of the confidence interval bracket. A histogram of the K_a_/K_s _distribution is shown (Figure 6). Following a method similar to Novaes *et al*. [19], we further classified genes with K_a_/K_s _< 0.15 to be under stabilizing selection, and K_a_/K_s _between 0.50 and 2.50 for diversifying selection, and compared the K_a_/K_s _distribution with the gene annotation results. This approach gave us 978 annotated contigs in stabilizing category and 923 annotated contigs in diversifying category. In both categories, the distribution of GO IDs by contigs was the highest for the GO class 'molecular function' with 103 unique GO IDs distributed among 508 (51.9%) contigs in stabilizing category and 103 GO IDs distributed among 448 (48.5%) contigs in diversifying category.

## Discussion

Previous reports of ESTs from non-model, yet ecologically and economically important organisms have been successfully sequenced and annotated using next generation sequencing [19-21]. Here, we have used 454 next-generation sequencing to generate the first transcriptome sequence data for big sagebrush, a key ecological species of the western North America. Similar to reports of other efforts, the assembled ESTs of big sagebrush were further analyzed to generate a functional characterization of the transcriptome and discover putative molecular markers (SSRs and SNPs). A surprisingly high level of nucleotide diversity was also found within individual assemblies of ESTs from big sagebrush accessions.

To generate a functional characterization of the big sagebrush transcriptome, we compared the contigs and singletons obtained from the combined assembly to peptides within the non-redundant protein database using BLASTx. The low number of matches (54 contigs) to *Artemisia annua *sequences is probably due to fewer number of *A. annua *sequences available in the NR database compared to species such as *Vitis vinifera*. We expect that the numbers of hits will substantially increase with the eventual publication and annotation of an *A. annua *and other *Artemisia *and Asteraceae genome sequences. A majority (69.8%) of the assembled sequences did not align with any peptide in the NR database, possibly indicating the presence of substantial number of novel genes in *A. tridentata *transcriptome and related taxa. Genes of unknown function are not unexpected, as the discovery of novel genes has been demonstrated in other EST sequencing projects within non-agricultural plant families [2,22].

Many of the contigs and singleton ESTs identified in this study are expected to have ecological and adaptive relevance. Previous studies relating sagebrush biochemistry to mule deer feeding preference suggest strong correlation between the composition and concentration of secondary metabolites, especially terpenoids, and mule deer preference of sagebrush [23,24]. We were able to identify many, but not all, of the genes coding enzymes involved in MVA, MEP, and phenylpropenoid pathways. The failure to detect all genes from these pathways could be explained by a lack of transcriptome coverage and/or by a lack of pathway documentation of these specific genes [25]. The detection of major enzymes involved in phenylpropanoid pathway in big sagebrush and variation within these pathways may aid in elucidating herbivore preferences and trade-offs between defense responses.

### Polymorphisms in A. tridentata ESTs

A large number of SNP and SSR markers were discovered and different subsets of SNPs were validated using Sanger amplicon sequencing of cDNA and genomic DNA, Illumina cDNA sequencing of ssp. *wyomingensis*, and sequence capture. We verified (Type 1) six of six tested SNPs using amplicon Sanger sequencing of individually selected PCR fragments. Additional verification (Sanger sequencing of next-generation sequencing results) was deemed unnecessary due to past experience in Arabidopsis [26], Amaranth [27,28], and cotton (Udall, personal communication) using this same conservative bioinformatic pipeline. These other studies verified 100% of 5 × more SNPs using Sanger re-sequencing of amplicons and demonstrated that they segregated in mapping populations such that genetic maps were reliably constructed. Similar to these other studies, a small number of genotypes (2) were used for SNP discovery in sagebrush ESTs. It was possible that the two individuals selected for EST sequencing could also represent minor alleles at a number of SNPs. Thus, the SSRs and SNPs that we report here represent DNA differences between individuals and differences between subspecies.

In our efforts to describe SNPs in big sagebrush, we have also quantified the number of SNPs that were due to subspecies differences and those that were due to individual differences. The high numbers of SNPs between individuals, apparent in the individual assemblies (of two individuals), in the validation using ssp. *wyomingensis*, and in the sequence capture assemblies (of four individuals) suggested significant amounts of nucleotide diversity between individual genomes of *Artemisia*. This evidence was supported by three findings. 1) When discriminating SNPs between ssp. *tridentata *and ssp. *vaseyana *were re-identified at a higher stringency than 90% (at 99%), 13% of the SNPs were not detected because of a single parameter requiring a degree of homogeneity among residues originating from a single DNA source. This suggests that both individuals used for EST sequencing contained a high number of heterozygous loci. 2) Using Illumina sequencing, only 36% and 44% of the SNP positions had both alleles detected in the ssp. *wyomingesis *samples respectively, where nearly all of the SNP positions were at least represented by one or the other allele. This indicated that both alleles of a significant number of the SNPs exist in a third *A. tridentata *subspecies, but a true polyploid hybrid of these the two diploid subspecies would contain both alleles of all SNPs. Thus, the ssp. *wyomingensis *samples used here were likely derived from different diploids and those individuals had significantly different genotypes than those used for EST sequencing. 3) Using sequence capture, only 54% of the 403 SNP positions were validated as discriminatory between ssp. *tridentata *and ssp. *vaseyana*, but 67% of the SNP positions had both bases detected. Thus, 13% of the sequence capture validated SNP positions also appeared to be heterogeneous (two nucleotides) within the collected individuals used for sequence capture. Indeed, a significant number of SNPs were found between individual plants within *A. tridentata *subspecies. Much of this nucleotide diversity at initially identified SNP loci could be at heterozygous loci, though we are careful not to describe it as such until allelism between nucleotide residues is fully established through segregation analysis. Recall that these EST sequences contain both coding and non-coding sequence (particularly the 3' UTR as the poly-A tail was used for priming the cDNA synthesis). A high level of nucleotide diversity in these coding and non-coding sequences is certainly plausible considering the very large effective population size of big sagebrush and wind-pollination strategy [29].

Given the high level of heterozygosity due to the out-crossing nature of big sagebrush populations [29], we expect that a large number of inter-subspecific SNPs and intra-subspecific SNPs could be used in conducting subspecies level association genetics studies. To date, little or no sequence of big sagebrush has been made publicly available, thus the SNPs reported here represent a starting point for such future population genetic studies of big sagebrush. While they may form the basis of future molecular studies, caution is needed because informative SNP comparisons will depend on the specific individuals selected for genetic analysis. Alternatively, our study suggests that a sequenced based approach to population genetics such as a population-wide genome reduction strategy [28] or amplicon analysis should be considered because of the expense required for assay development and their potential use in few, specific *A. tridentata *individuals. Such an approach would avoid extrapolation of our putative SNPs specific to these individuals to a larger population of individuals (*e.g. *subspecies' specific SNPs that were likely due to genetic variation between individuals) by generating accession-specific data for each newly sequenced accession. Implementation of such study among spatially distributed big sagebrush populations would 1) enlighten our understanding of natural selection on genes and gene complexes controlling adaptive traits, and the evolution of these trait-linked loci and 2) provide relatedness metrics between natural populations of these subspecies and their hybrid zones. Though we briefly touched on these questions by using independent genotypes for SNP validation, these questions are out of the scope of this particular study that aims to primarily characterize EST sequences of big sagebrush and provide insight regarding the origins of ssp. *wyomingensis.*

Regarding the discovered SSRs, we were surprised to find that all SSR repeat motif types detected were much more abundant in ssp. *tridentata *compared to ssp. *vaseyana*. The reduced levels of SSR in ssp. *vaseyana *ESTs compared to ssp. *tridentata *could be due to differential gene expression since different loci were sampled with our non-replicated experimental design. While leaves from both plants were harvested at the same time in common garden, phenological differences between the subspecies might have caused differences in expression levels and thus, changes in the number and types of detected SSRs. While gene expression could explain some of the differences, many such EST-SSRs have been found to be reliable molecular markers in other species [22,30-33] and they represent hypothetical (*i.e. *testable) genetic divergences between the subspecies.

### K_a_/K_s _and gene evolution in big sagebrush

The ratio of synonymous and non-synonymous mutations between sspp. *tridentata *and *vaseyana *suggest possible selection pressure resulting in the maintenance of subspecies divergence, as similar trends have been observed in various organisms [34-37]. Since natural selection shapes phenotypes and genotypes in favor of adapted traits, the K_a_/K_s _ratio of less than 1 for a large number of contigs could be a result of either stabilizing or diversifying selection within both subspecies, depending upon the magnitude of the ratio. Or if divergence times are very recent, it could also be the hallmark of purifying selection on the adapted common ancestor of these two subspecies. For example, Contig_29840 (K_a_/K_s _= 0.106) was annotated for 'aquaporin' protein. Considering that big sagebrush grows in variety of soils and arid plains, valleys and foothills of mountains, the importance of aquaporin proteins in water balance is critical and the genes coding for aquaporin proteins could have been under stabilizing selection. A formal investigation of molecular evolution within these species (with a proper outgroup) would place selection pressure relative to species divergence.

### Exploring the inter-subspecies hybridization hypothesis

Hybridization can be of great importance to the ecological adaptation and subsequent evolution of offspring because of the novel genetic recombination and spatial selection [38-40]. Generally, allopolyploid formation is considered to have arisen through hybridization between unreduced gametes [41-43]. Several studies have been conducted on hybrid populations formed from *A. t. *ssp. *tridentat*a and *A. t. *ssp. *vaseyana *to investigate hybridization events. Generally, these hybrid populations are formed in a narrow zone between the two ecotypes [29,44-47]. In this study, we did not select a tetraploid ssp. wyomingensis along with diploid representatives of its two neighboring ssp. *tridentata *and ssp. *vaseyana *populations. Instead, selected ssp. *tridentata *and ssp. *vaseyana *accessions were chosen for EST sequencing based on penetrance of specific, subspecies morphological markers (*i.e. *trueness to type). Thus, variation at SNP loci for the diploid-tetraploid comparison is a mixture of individual variation, variation within inter-mating populations, and variation between subspecies in this study. Based on the number of Illumina reads that actually did map to discriminating SNPs between sspp. *tridentata *and *vaseyana*, the tetraploid ssp. *wyomingensis *samples appeared to contain both alleles for a large number of loci (251/695 Montana; 458/1,039 Utah). The presence of both alleles at approximately one-third of the loci suggests that ssp. *wyomingensis *either originated as an allotetraploid from a hybridization event of 2 n gametes between sspp. *tridentata *and *vaseyena *or formed as a autopolyploid from both diploid subspecies with subsequent hybridization. Since allopolyploids have been reported between diploids and tetraploids of ssp. *tridentata *and ssp. *vaseyena *[9,29,46,48], a similar scenario is plausible for the origin of ssp. *wyomingensis*. A focused genetic study within and between putative hybrid zones of big sagebrush is needed to further elucidate the origins and reproducibility of hybridization processes involved in ssp. *wyomingensis *formation. If tetraploid recurrence is a common feature of ssp. *wyomingensis*, perhaps only populations of ssp. *tridentata *and ssp. *vaseyana *need active management during environmental conversation of wildlands because a tetraploid hybrid between the two locally adapted accessions could be expected to form and repopulate geographic zones between the diploid subspecies.

## Conclusions

This study is the first of its kind to perform transcriptome sequencing of big sagebrush subspecies, generating large selections of genetic resources (EST sequences, SNP markers and microsatellites) for this ecologically important group of range and forest plants. The EST sequences were annotated to identify putative gene functions, and select genes involved in putative terpenoid and coumarin synthesis were bioinformatically identified. The distribution of SNPs among *A. tridentata *subspecies and the estimation of depth and divergence of mutations provide insights about the magnitude of neutral divergence and natural selection between these subspecies, and a foundation of sequence references for future population genomic and functional genetic studies. The cost-effective, rapid and reliable way of obtaining nuclear sequences through transcriptome sequencing also provided insights on gene divergence and marker development in big sagebrush. Future studies integrating common garden, provenance and reciprocal transplantation of defined genetic stocks with this genomic information will immeasurably add to our understanding patterns of genes and their roles in adaptive traits among big sagebrush populations.

## Methods

### Plant materials and RNA extraction

Young leaves from two subspecies of big sagebrush, *A. tridentata *ssp. *tridentata *and *A. tridenata *ssp. *vaseyana*, were harvested from plants growing in USDA Shrub Lab greenhouse in Provo, UT for 454-pyrosequencing (Additional File 5). The plants were grown from seeds collected in their natural habitat near Park Valley, UT. The leaves were flash frozen in liquid N_2 _and stored in -80°C until further use. RNA extraction was performed using approximately 0.1 g of frozen leaf tissue, following a modified hot borate procedure [49]. The extracted RNA was analyzed for quality and quantified using Agilent 2100 Bioanalyzer (Agilent Technologies, Foster City, CA) before using for cDNA synthesis.

### cDNA library preparation for 454-pyrosequencing

cDNA was created using 1 μg of total RNA using the SMART cDNA synthesis kit (Clontech Laboratories, Inc., Mountain View, CA), but the cDNA synthesis primer for first strand synthesis was replaced by a modified oligo-dT primer (5'-AAGCAGTGGTATCAACGCAGAGTCGCAGTCGGTACTTTTTTCTTTTTTV-3') [50]. The poly-T stretch in the primer is broken by inserting a Cytosine to minimize the potential sequencing problems due to the presence of a long ploy-A homopolymer stretch. The cDNA library was normalized using the Trimmer Kit (Evrogen, Moscow, Russia) to limit redundant sequencing of highly expressed genes. We did not directly test normalization values since so few transcripts were known for big sagebrush prior to this report. The normalization control included with the Trimmer Kit was reduced in copy number as expected. Because this control was normalized as expected, we assumed that a similar normalization of highly expressed genes also occurred in our two sagebrush samples. Adaptors ligation and single strand selection were done as described in the GS FLX Titanium General Library Preparation Kit (Roche, Branford, CT) with modifications. One half-plate was sequenced for each subspecies at the Brigham Young University DNA sequencing center, Provo, UT.

### Illumina sequencing of A. t. ssp. wyomingensis and SNP mapping

Leaves were harvested from two young *A. t. *ssp. *wyomingensis *plants growing in USDA Shrub Lab greenhouse in Provo, UT. The plants were grown from seeds collected in their natural habitat in two different states - Montana and Utah. Geographic information on sampled individuals is provided in Additional file 5. Tetraploid confirmation was conducted on a Partec PAII flow cytometer. Leaves from each plant along with a known *A. tridentata *ssp. *tridentata *diploid standard were finely chopped in a buffer and then nuclei were stained with DAPI solution (CyStain UV Precise P, Partec). Total RNA was harvested and quantified in the same manner as mentioned above. The RNA was processed for sequencing following directions in the Illumina mRNA Sequencing Sample Prep Guide (part #1004898 rev. D., Illumina, Inc., San Diego, CA), with the addition of custom barcoded adapters designed for the paired-end sequencing process [51]. The quality of the libraries was validated using the Agilent 2100 Bioanalyzer. The prepared libraries of the ssp. *wyomingensis *individuals were multiplexed in approximately equal concentrations and sequenced in two separate runs (one single end 80 bp run, and a second paired end 80 bp run) on the Illumina Genome Analyzer at the Oregon State University Center for Gene Research and Biocomputing, Corvallis, OR. Pooled libraries were loaded onto one lane of an Illumina Genome Analyzer II at 5 pM concentration. Cluster generation and sequencing used Illumina version 3.0 reagents, and image acquisition and base calling used the Illumina pipeline version 1.5. These Illumina sequences were used only to verify in ssp. *wyomingensis *the SNP loci detected on the combined assembly of sspp. *tridentata *and *vaseyana *obtained from 454 sequences.

Bowtie [52] was used to sort and align the Illumina reads to the 'reference' combined assembly, with no gaps and allowing a single base mismatch. The mismatch alignment results were compared to the SNPs obtained from the combined assembly of two subspecies, and the output was parsed so that the SNPs were covered by 1 or more ssp. *wyomingensis *reads. For confident SNP calling, we required only the SNPs covered by coverage of 20 × or more be counted; and 10% of all the reads overlapping a SNP were required to be of a particular variant in order to avoid SNP detection that could have resulted from sequencing errors.

### EST sequence de novo assembly and annotation

A combined *de novo *assembly of the sequences from both subspecies was performed using CLC Genomics Workbench Version 3.7.1 (CLC bio, Cambridge, MA). The sequence ends were trimmed to remove the barcodes added during library preparation, and any sequence shorter than 50 bp was not included in building the assembly. The mismatch cost for the nucleotides was set at 2 while both the insertion cost and deletion cost for nucleotides in the reads were set at 3. The length fraction and the similarity of the sequences were set at 0.5 and 0.9, respectively. Any conflicts among the individual bases in the reads were resolved by voting for the base with maximum number of repetitions. A minimum read length of 200 bp was set for an assembled sequence to be counted as a contig. Identical parameters were also used to create individual assemblies from both subspecies. Homologies of the contigs and singletons were identified by comparing against the NCBI NR protein database using BLASTx with cut-off e-value of 1e^-15^. The blast results were imported into Blast2GO Version 2.4.2 for mapping the consensus sequences into GO terms. To summarize the distribution of the sequences into GO terms of three main categories - biological processes, cellular components and molecular functions, GO annotations were formatted for input into the GOSlim program [53]. The consensus sequences from combined assembly of both subspecies were also searched against the Pfam-A database using the HMMER software Version 3.0 [54]. Protein sequences generated by ESTScan Version 2-2.1, using the *Arabidopsis thaliana *gene sequences as the reference matrix, were used for this purpose.

### Polymorphism detection

SNPs were identified between the subspecies using the Perl script used by Maughan *et al*. [28]. For the nucleotides to be counted as a SNP, the following parameters were required: 1) the coverage depth of the read at the SNP was ≥ 8; 2) the minimum frequency of the minor allele was 20%; and 3) within each possible nucleotide at that SNP position, ≥ 90% of its bases at the SNP position are from a single subspecies (*i.e. *'heterozygosity' at 10%). For example, a G↔A SNP would be included in the list of SNPs at coverage of 100×, if, out of 100 aligned sequences, 80 sequences came from one subspecies with at least 72 sequences calling for a G, and 20 sequences came from another subspecies with at least 18 sequences calling for an A at the SNP position. Primers for SNP validation were designed using Primer3 [55].

Perl script MISA (MIcroSAtellite identification tool, [56]) was also used to identify SSRs in the assembled consensus sequences. Motif repeats between di and deca-units were searched for in the assembly of each subspecies. For repetitive nucleotide stretches to be counted as an SSR unit, di-nucleotide repeats had to be present in frequency of 7 units, tri-nucleotide to octa-nucleotide repeats in frequency of 5 units and nona- and deca-nucleotide in frequency of 4 repeats. The interruption distance among any type of compound SSR units was set at 100 bp. Using the same parameters used by MISA to detect SSRs in each subspecies dataset, the program SSR locator [57] was used to detect SSRs and design primers in the combined assembly of sequences of both subspecies. An additional, custom Perl script was written to bioinformatically distinguish polymorphic SSR loci between the two subspecies ('true' SSRs). An SSR would be counted as a polymorphic SSR if an indel of the whole repeat motif and/or few bases in the motif was detected in the consensus sequences of each subspecies.

### Polymorphism Validation

Sanger re-sequencing of both subspecies cDNA validated consensus sequences containing SNPs and the SSRs. Fifteen putative SNP loci and SSR loci were randomly selected for PCR amplification. We selected 11 SNPs with transition mutation and 4 with transversion mutations for PCR amplification followed by SNP validation by re-sequencing. For validation purpose, we selected equal number of transitions and transversions (3 each, 6 total). The SSR primers were designed to amplify 4 di-, 5 tri-, and 4 tetra-, 1 penta- and 1 hexanucleotide SSRs of varying lengths. Two tetraploid ssp. *tridentata *individuals, two diploid and one tetraploid ssp. *vaseyana *individuals and two tetraploid ssp. *wyomingensis *individuals from geographically divergent sources were used for SNP and SSR marker evaluation in genomic DNA. Geographic information on these individuals is provided in Additional File 5. The following settings were followed for PCR amplification of both SNP and SSR loci: 2 mM MgCl_2_, 1 × PCR buffer, 0.2 mM dNTPs, 0.6 μM of each primer, 1 U *Taq *polymerase and dH_2_O to a final reaction volume of 10 μl ran in the following thermal profile: 94°C 3 min, 35 × (94°C 30 s, Tm depending upon the primers used 45 s, 72°C 1 min) and final extension of 72°C 10 min. PCR reactions of both SNP loci and SSR loci were cleaned using Qiaquick PCR Purification kit (Qiagen, Valencia, CA), and then mixed with appropriate primers prior to sequencing. Fragments were sequenced with an ABI 3730xl DNA analyzer at the University of Wisconsin Biotechnology Center, Madison, WI. The primers used for SNP and SSR validation are listed in Additional File 5.

SNPs and SSRs were validated by sequencing 369 genes containing 572 SNPs in sspp. *tridentata *and *vaseyana *using sequence capture. DNA extractions were made of NVT-2 and UTT-2 (ssp. *tridentata *collected from 39°29'22'' 117°85'17'' and 38°30'60'' 109°38'76'', respectively) and UTV-1 and UTV-3 (ssp. *vaseyana *collected from 39°34'13'' 111°52'21'' and 38°34'13'' 109°21'73'', respectively) RNA baits (120-mer) of the target genes were synthesized after the contigs obtained from the combined assembly. The DNA libraries for bait hybridization were prepared using GS FLX Titanium Rapid Library Preparation Kit (Roche, Branford, CT, USA). The baits were then hybridized to the library and the capture sequences were prepared for 454-sequencing following the MYselect protocol (MYcroarray, Ann Arbor, MI, USA). The captured DNA libraries were pooled and sequenced at the Brigham Young University sequencing center, Provo, UT. Using 0.95 sequence similarity, the capture reads were mapped to the reference contigs in order to place individual reads at previously called SNPs and SSRs using Genomics Workbench 4 (CLCBio, Aarhus, Denmark). Custom Perl scripts were used to assess SSR and SNP coverage and validate the presence of SNP bases in distinct DNA samples of sspp. *tridentata *and *vaseyana*.

### Analysis of synonymous and non-synonymous mutations

To find the changes in amino acid sequences due to single nucleotide mutations, we aligned sspp. *tridentata *and *vaseyana *contigs that had their coding frame predicted using ESTScan. The proportion of non-synonymous to synonymous mutations (K_a_/K_s_) was calculated for each contig, following the Jukes-Cantor corrected model of substitution using Bioperl modules [58]. We however modified the Jukes-Cantor equation by adding one unit to both non-synonymous and synonymous substitutions in order to obtain a valid K_a_/K_s _estimation in instances where either type of substitution was absent. Without this modification, we would have obtained K_a_/K_s _value equal to zero for genes with no observed non-synonymous substitutions, regardless of their K_s _values. Similarly, genes without any synonymous substitutions would have undefined K_a_/K_s_.

## Authors' contributions

PB prepared the cDNA libraries, assembled the EST sequences, carried out the GO annotation, developed the SNP and SSR markers, and drafted the manuscript. BAR helped with the collection of plant materials from the field for 454 and Illumina sequencing and edited the manuscript. JCP sorted the Illumina sequences, and mapped the ssp. *wyomingensis *reads to 454 contigs to identify SNPs. RCC prepared the ssp. *wyomingensis *transcriptome for Illumina sequencing and supervised the sequencing. JU conceived this study, provided bioinformatic training, and drafted the final manuscript. All authors read and approved the final manuscript.

## Supplementary Material

Additional file 1 **Distribution of protein domain vs number of contigs**. The number of contigs on Y-axis represents total number of contigs that had a match against a protein domain. Only the top 25 most common domains (of 3065 domains found) are illustrated in the figure. Click here for file

Additional file 2 **The distribution and sequences of putative sagebrush homologs of enzymes involved in terpenoid and coumarin synthesis pathways**. The data consists of contigs of each subspecies annotated as terpenoid and coumarin pathway enzymes, as well as the contigs that resulted from combined assembly. The nucleotide sequences of the putative genes (contigs from the combined assembly) have also been included in the file. Click here for file

Additional file 3 **A list of contigs containing discriminatory SNPs between ssp. tridentata and ssp. vaseyana including contig name, SNP position, base for each subspecie, read count per base, flagged contigs with >13 SNPs, and SNPs that were found to be heterogeneous when the parameter of homogeneity was raised to 99%**. Click here for file

Additional file 4 **Additional details of SSRs including frequencies of di- and tri-nucleotide repeats**. Click here for file

Additional file 5 **Details of SNP and SSR primers used for polymorphism validation and the list of big sagebrush individuals used during the project**. Click here for file

Additional file 6 **Results for SNP validation during sequence capture**. Click here for file
