# Supplementary material for: Transcriptome characterization and polymorphism detection between subspecies of big sagebrush (Artemisia tridentata)
Source: BMC Genomics. 2011 Jul 18;12:370. doi: 10.1186/1471-2164-12-370 (PMC3150299; doi:10.1186/1471-2164-12-370)

Supplemental File S1 – Distribution of protein domain vs number of contigs

The number of contigs on Y-axis represents total number of contigs that had a match against a protein domain. Only the top 25 most common domains (of 3065 domains found) are illustrated in the figure.

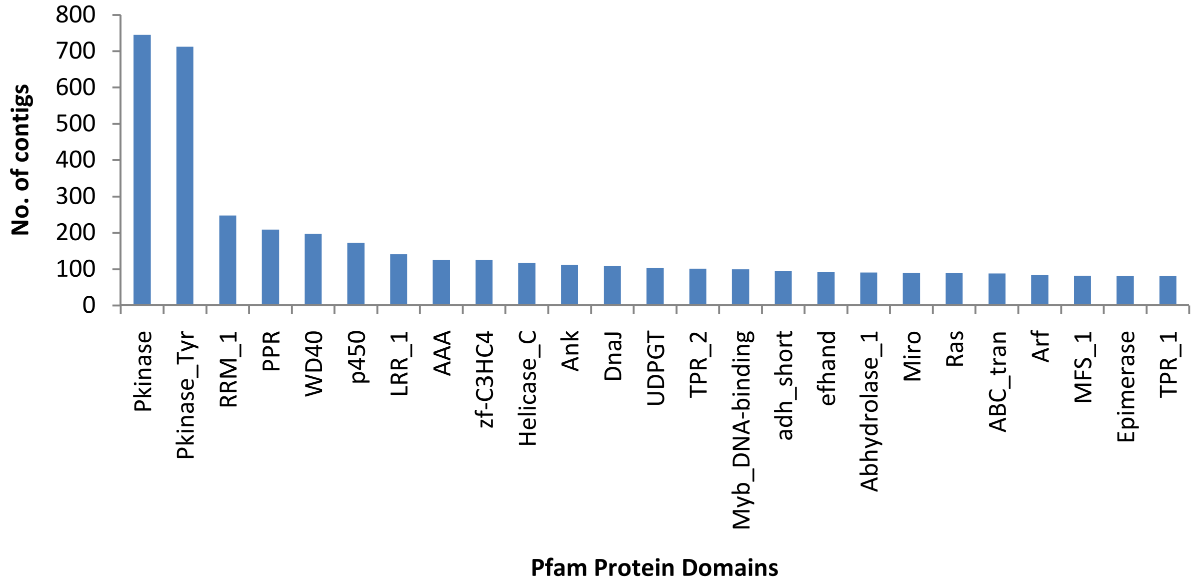

Supplement: Additional file 1 — Distribution of protein domain vs number of contigs. The number of contigs on Y-axis represents total number of contigs that had a match against a protein domain. Only the top 25 most common domains (of 3065 domains found) are illustrated in the figure. [file 1471-2164-12-370-S1.PDF]
