# Supplementary material for: Transcriptome characterization and polymorphism detection between subspecies of big sagebrush (Artemisia tridentata)
Source: BMC Genomics. 2011 Jul 18;12:370. doi: 10.1186/1471-2164-12-370 (PMC3150299; doi:10.1186/1471-2164-12-370)
Supplement: Additional file 4 — Additional details of SSRs including frequencies of di- and tri-nucleotide repeats. [file 1471-2164-12-370-S4.DOCX]

**Additional File 5: Frequencies of different repeat motifs of di- and trinucleotide repeats in EST-SSRs from *A. t.* ssp. *tridentata* (*A.t.t.*) and ssp. *vaseyana* (*A.t.v.*)**

| **Repeat Motif** | **Repeat Number** | | | | | | | | | | | | **Total** | |
| --- | --- | --- | --- | --- | --- | --- | --- | --- | --- | --- | --- | --- | --- | --- |
|  | ***A.t.t.*** | ***A.t.v.*** | ***A.t.t.*** | ***A.t.v.*** | ***A.t.t.*** | ***A.t.v.*** | ***A.t.t.*** | ***A.t.v.*** | ***A.t.t.*** | ***A.t.v.*** | ***A.t.t.*** | ***A.t.v.*** | ***A.t.t.*** | ***A.t.v.*** |
|  | **5** | | **6** | | **7** | | **8** | | **9** | | **≥10** | |  |  |
| **AC/GT** | - | - | - | - | 89 | 11 | 27 | 1 | 11 | 1 | 25 | 6 | 152 | 19 |
| **AG/CT** | - | - | - | - | 14 | 0 | 6 | 1 | 6 | 0 | 6 | 3 | 32 | 4 |
| **AT/AT** | - | - | - | - | 1 | 4 | 1 | 0 | 0 | 0 | 0 | 0 | 2 | 4 |
| **AAC/GTT** | 69 | 26 | 21 | 10 | 16 | 5 | 4 | 3 | 1 | 0 | 4 | 1 | 115 | 45 |
| **AAG/CTT** | 68 | 37 | 37 | 16 | 12 | 3 | 3 | 3 | 2 | 3 | 5 | 2 | 127 | 64 |
| **AAT/ATT** | 21 | 9 | 15 | 8 | 5 | 4 | 1 | 0 | 0 | 0 | 0 | 1 | 42 | 22 |
| **ACC/GGT** | 82 | 58 | 42 | 33 | 7 | 9 | 3 | 4 | 0 | 1 | 0 | 0 | 134 | 105 |
| **ACG/CTG** | 20 | 7 | 8 | 5 | 4 | 1 | 2 | 0 | 0 | 1 | 0 | 0 | 34 | 14 |
| **ACT/ATG** | 50 | 41 | 22 | 16 | 12 | 2 | 8 | 3 | 4 | 2 | 4 | 0 | 100 | 64 |
| **AGC/CGT** | 22 | 19 | 4 | 12 | 4 | 3 | 1 | 1 | 2 | 1 | 0 | 1 | 33 | 37 |
| **AGG/CCT** | 21 | 12 | 9 | 6 | 4 | 3 | 1 | 0 | 0 | 0 | 0 | 0 | 35 | 21 |
| **AGT/ATC** | 68 | 39 | 28 | 21 | 11 | 5 | 7 | 2 | 1 | 2 | 4 | 3 | 119 | 72 |
| **CCG/CGG** | 10 | 2 | 0 | 0 | 0 | 0 | 0 | 0 | 0 | 0 | 0 | 0 | 10 | 2 |
